# Single-cell analysis of lipopolysaccharide-mediated heterogeneity in *Escherichia coli* adhesion and mechanics

**DOI:** 10.1007/s00203-025-04529-3

**Published:** 2025-10-18

**Authors:** Dariusz Laskowski, Janusz Strzelecki

**Affiliations:** 1 Department of Microbiology, Faculty of Biological and Veterinary Sciences, Nicolaus Copernicus University in Toruń, Lwowska 1, 87-100 Toruń, Poland; 2 Institute of Physics, Faculty of Physics, Astronomy and Informatics, Nicolaus Copernicus University in Toruń, Grudziądzka 5, 87-100 Toruń, Poland

**Keywords:** Phenotypic heterogeneity, Lipopolysaccharides, Bacterial adhesion and mechanics, Atomic force microscopy, Single-cell analysis

## Abstract

Bacterial populations that exhibit phenotypic subgroups of cells with different biophysical properties are better adapted to colonize diverse surfaces, thereby facilitating survival and dissemination in varied environments. In this study, we applied atomic force microscopy (AFM) to investigate lipopolysaccharide (LPS)-mediated heterogeneity in the adhesion and mechanics of individual *Escherichia coli* ATCC 25922 cells within a clonal population. Our results, based on both single-cell and population level analyses, revealed that partial LPS removal by ethylenediaminetetraacetic acid (EDTA) substantially altered the bacterial cell surface, rendering it smoother and featureless. EDTA-induced disorganization of the outer membrane not only diminished both adhesion forces and cell elasticity but also markedly reduced the structural diversity of the cell envelope, thereby decreasing cell-to-cell heterogeneity. Notably, the population no longer exhibited cells with a strongly adherent and stiff phenotype. Our findings indicate that the structural and chemical diversity of the outer membrane, primarily conferred by LPS, is a key determinant of phenotypic heterogeneity, with important implications for bacterial adhesion, environmental adaptation, and responses to antimicrobial stressors. Overall, this study emphasizes the essential role of LPS in modulating bacterial surface interactions and mechanical behavior, and demonstrates the utility of AFM-based force spectroscopy with colloidal probes as a quantitative platform for analyzing single-cell variability.

**Supplementary Information:**

The online version contains supplementary material available at 10.1007/s00203-025-04529-3.

## Introduction

Microbial populations are highly complex and heterogeneous systems, comprising phenotypic subgroups of cells that exhibit differences in growth rate, motility, biofilm formation, antibiotic resistance, pathogenicity, and virulence (Alsteens et al. 2013; Perrier et al. 2019; Schröter and Dersch 2019; Zhou et al. 2025). Even genetically identical bacteria often exhibit marked phenotypic heterogeneity at the single-cell level, which can emerge rapidly after cell division (Heyse et al. 2019; Pani and Mohapatra 2024). Additionally, the bacterial cell envelope is highly heterogeneous, spatially organized, and exhibits species-specific properties that vary with growth phase and environmental conditions (Araújo et al. 2010; Alsteens et al. 2013). Diversity within clonal populations provides a selective advantage under environmental perturbation, thereby increasing population level fitness (Davis and Isberg 2016). Bacteria that exhibit varied biophysical properties, such as rigidity and adhesion, can efficiently colonize virtually all natural and synthetic surfaces, which is essential for their survival in environments such as soil, water, and host organisms (Chen et al. 2014; Fux et al. 2023). Furthermore, shortly after the initial attachment, bacteria enter an adherent state that modulates adaptive responses to environmental conditions. Prolonged adhesion ultimately leads to biofilm formation, wherein cells gain protection against antibacterial agents, environmental stressors, and the host immune system (Carniello et al. 2018; Alam et al. 2019).

The outer membrane (OM) is a unique and essential structure in Gram-negative bacteria that serves multiple functions (Storek et al. 2024). However, only recent studies have highlighted its substantial contribution to cellular stiffness and mechanical strength (Rojas et al. 2018; Kiss et al. 2021; Sun et al. 2022). The mechanical integrity of the bacterial cell is determined not only by the elastic properties of the peptidoglycan (PG) but also by the viscoelastic OM (Vadillo-Rodriguez and Dutcher 2009; Rojas et al. 2018; Araújo et al. 2019) and its connection to the PG mediated by Braun’s lipoprotein (Mathelié-Guinlet et al. 2020). Chemical or genetic alterations to the OM markedly enhance cell envelope deformation under mechanical loading (Rojas et al. 2018). Upon contact with a surface, bacteria are subjected to adhesion forces that induce mechanical stress and minor deformation of the cell envelope (Berne et al. 2018), which increases the contact area and thereby enhances adhesion strength (Chen et al. 2014; Carniello et al. 2018). Furthermore, viscoelastic deformation of the cell envelope, along with associated changes in adhesive proteins and appendages, enable surface sensing in bacteria and may function as a mechanosensitive signal that triggers downstream cellular responses, including enhanced production of adhesion factors (Berne et al. 2018; Viela et al. 2020). Thus, bacterial adhesion is a complex process involving both specific and non-specific interactions, determined by the physicochemical and mechanical properties of the cell, the substratum, and the surrounding environment (El-Kirat-Chatel et al. 2020; Zheng et al. 2021; Parreira and Martins 2021).

To date, little research has examined the occurrence and functional implications of phenotypic heterogeneity in bacterial adhesive and mechanical properties at the single-cell level, particularly in relation to the role of lipopolysaccharides (LPS). Existing studies have primarily focused on the role of LPS in bacterial adhesion (Nakao et al. 2012; Saini and Wood 2020; Aleksandrowicz et al. 2021) and cell stiffness (Rojas et al. 2018; Kiss et al. 2021; Sun et al. 2022), as well as on the structural and functional characteristics of the outer membrane (Lu et al. 2011; Paracini et al. 2022; Storek et al. 2024). Much of this research has focused on uncovering the drivers of diversity, particularly at the molecular level (Avery 2006; Carlquist et al. 2012; Martins and Locke 2015; Davis and Isberg 2016; Heyse et al. 2019; Fux et al. 2023). Despite increasing evidence of variability at the single-cell level, many studies rely on averaged measurements, which mask physiologically relevant heterogeneity. Conventional atomic force microscopy (AFM) typically probes multiple points on the bacterial surface, providing information on local surface heterogeneity or yielding averaged values. Alternatively, single-cell AFM approaches often analyze only a small number of cells (typically 3 to 10), limiting their capacity to detect population level heterogeneity. In general, data collected from all cells are analyzed collectively, and the contributions of individual cells are usually not discussed in detail (Abu-Lail and Camesano 2003; Kang and Elimelech 2009; Carlquist et al. 2012; Zeng et al. 2014; Carniello et al. 2018). Therefore, the impact of outer membrane perturbations, particularly LPS removal, on biophysical heterogeneity across bacterial populations remains poorly understood. Nonetheless, substantial structural diversity has been observed on bacterial surfaces, resulting in significant measurement variability that suggests the presence of distinct subpopulations. For instance, El-Kirat-Chatel et al. (2017) reported occasional outliers, despite similar behavior among most cells from independent cultures. Mittelviefhaus et al. (2019) observed significant variability in hydrophobic adhesion forces among bacterial strains isolated from the leaves. Likewise, Park and Abu-Lail (2011) demonstrated that adhesion energies varied among individual cells, regardless of whether they originated from the same or different cultures. However, the mechanical properties of single cells in a pure culture remain poorly characterized. Only a few studies have investigated the role of bacterial elasticity in determining adhesion propensity. Tamayo et al. (2020) identified subpopulations within a single culture that exhibited differences in stiffness and adhesion energy, suggesting that reduced cell elasticity may enhance bacterial adhesion. Similarly, Chen et al. (2014) reported that minor deformations of the bacterial cell wall increase long-range adhesion forces. In contrast, Carniello et al. (2018) argued that adhesion forces exert limited influence on cell deformation. The relationships between elasticity and adhesion appears to be species-specific and strongly dependent on the deformation model applied in data analysis. In this context, single-cell biophysical techniques such as AFM-based force spectroscopy are particularly valuable.

In this study, we applied atomic force microscopy to investigate the role of lipopolysaccharides in generating heterogeneity in the adhesive and mechanical properties of individual *Escherichia coli* cells within a clonal bacterial population. We hypothesized that partial removal of LPS through ethylenediaminetetraacetic acid (EDTA) treatment would reduce cell-to-cell heterogeneity by homogenizing outer membrane organization and, consequently, the biophysical properties of individual cells. We used force spectroscopy with a colloidal probe instead of a standard conical tip to evaluate cell stiffness and bacteria-surface adhesion forces across the entire cell surface. This approach minimized the influence of known surface diversity and allowed us to focus on heterogeneity among cells within the population. We assessed how LPS removal modulates diversity in biophysical properties by quantifying cellular variability using a heterogeneity index. Additionally, single-cell measurements were compared with bacterial adhesion and aggregation assays to evaluate the relationship between single-cell and population level behaviors. This study builds upon our previous findings, which revealed substantial heterogeneity in *E. coli* populations (Laskowski et al. 2021).

## Materials and methods

### Bacterial strain

*Escherichia coli* ATCC 25922 was stored at −80 ° C, revived on Luria-Bertani (LB) agar (BD Difco, USA) and cultured in Luria-Bertani (LB) broth (BD Difco, USA) for 24 h at 37 ° C with shaking at 150 rpm.

### Removal of lipopolysaccharides from the bacterial membrane

Lipopolysaccharides were released from bacterial surface using ethylenediaminetetraacetic acid, as described by Chen et al. (2009), with some modifications. Bacterial culture was centrifuged at 2151 × g for 5 min at 24 ° C, and cell pellets were washed with Milli-Q water. Cells were than resuspended in a 100 mM EDTA solution (pH 8.0, Sigma-Aldrich, Inc., USA) and incubated at 37 ° C for 30 min with gentle shaking (vertical swinging motion at 20 rpm). The cells were then recentrifuged under the same conditions, washed twice with Milli-Q water, and resuspended in 0.01 M phosphate buffer (pH 7.0, Sigma-Aldrich, Inc., USA) for analysis.

AFM imaging was performed to verify that EDTA treatment did not visibly compromise the integrity of the *E. coli* cell envelope through membrane rupture or lysis, which could indicate loss of viability. The treated bacteria retained their normal cylindrical shape, with no visible pores or structural ruptures. Cells undergoing division were also observed, further supporting that viability was preserved under the treatment conditions.

### Bacterial sample preparation for atomic force microscopy

Bacteria were immobilized on gelatin-coated glass surfaces (Sigma-Aldrich, Inc., USA) using a procedure adapted from Louise Meyer et al. (2010). Cells were centrifuged at 2151 × g for 5 min at 24 ° C, washed twice with Milli-Q water, and resuspended. The suspension was adjusted at 10^6^ CFU/ml and deposited on gelatin-coated slides for 30 min. Loosely attached cells were removed by rinsing thoroughly with 0.01 M phosphate buffer (pH 7.2, Sigma-Aldrich, Inc., USA). All samples were kept in 0.01 M phosphate buffer (pH 7.2, Sigma-Aldrich, Inc., USA) for force spectroscopy analysis or washed in Milli-Q water and nitrogen-dried prior to AFM imaging. All single-cell measurements were conducted in two independent bacterial cultures.

### AFM imaging

AFM imaging was performed on an Agilent 5500 AFM (Agilent Technologies, Inc., USA) using a PPP-NCST-20 cantilever (NanoAndMore USA Corp., USA) with a resonant frequency of approximately 160 kHz in air. Images were acquired in intermittent contact mode. The scan rate and pixel resolution were 1 line/s and 512 × 512 pixels, respectively. Topography images were flattened using a zero-order set to remove scan line misalignment (each line was individually fitted to the center of the data) and used to measure surface roughness (Fig. S1). Surface roughness was quantified as the root mean square average of the selected area on the bacterial surface, using Gwyddion software 2.68 (Nečas and Klapetek 2012). At least two distinct areas, each with a fixed size of 400 × 400 nm^2^, were analyzed on ten bacterial cells.

### Preparation of colloidal probes

Colloidal probes were prepared following a protocol adapted from Beaussart et al. (2013), with some modifications. Triangular-shaped tipless MLCT-O10 cantilevers (Bruker Corp., USA) were cleaned with a UV lamp (253 nm emission, NBV 2 × 30 P, Ultra-Viol, Poland) for 30 min in a biological safety cabinet, rinsed with Milli-Q water, and dried in a vacuum (1 mbar) for about 15 min. Using an Agilent 5500 AFM connected to a Nikon Eclipse Ti-E inverted microscope (Nikon Corp., Japan), the cantilever end was immersed in a thin layer of UV-curable adhesive (6020, Drei Bond GmbH, Germany) spread on a glass slide and brought into contact with a silica microsphere (SiO_2_, 6.95 μm diameter, microParticles GmbH, Germany). The colloidal probe was then cured under a UV lamp for 15 min.

### Force spectroscopy of single-cells

AFM measurements were performed at room temperature (22 ° C) in 0.01 M phosphate buffer (pH 7.2, Sigma-Aldrich, Inc., USA) using an Agilent 5500 AFM mounted on a Nikon Eclipse Ti-E inverted microscope. Prepared cantilevers were calibrated prior each experiment by the thermal noise method (spring constant was 0.024 ± 0.004 N/m). Isolated, viable cells were selected for force spectroscopy measurements in contact mode. At least four force-distance curves were collected per cell from approximately 25–30 bacterial cells, using a maximum loading force of 250–500 pN, with a contact time of 100 ms and at constant retraction speed of 1 μm/s. Control force curves were acquired on bacteria-free area before and after proper measurements to confirm that the AFM probe was not contaminated by bacterial biopolymers. A contaminated probes were replaced. Force spectroscopy data were analyzed in SPIP software 6.4.2 (Image Metrology, Denmark). Adhesion force and rupture distance were calculated as previously described (Laskowski et al. 2018).

The Young’s modulus was determined from the force-indentation curves fitting using the Sneddon (sphere) model (Sneddon 1965). The AFM colloidal probe was modeled as a sphere with a radius of 3.475 μm and a Poisson ratio of 0.5 (incompressible material). Elasticity analysis was performed using AtomicJ version 2.3.1, an open-source software (Hermanowicz et al. 2014).

### Heterogeneity index

Heterogeneity in the adhesive and elastic properties of bacterial cells was quantified using the heterogeneity index (HI), calculated as described by Park and Abu-Lail (2011). HI was defined as the ratio of the standard error (SE) of the mean obtained for each individual cell to the maximum SE observed among all analyzed cells (the most heterogeneous cell). The index was expressed as a percentage. Intragroup HI reflects variability among measurements within individual cells, while intergroup HI described heterogeneity between experimental groups (control vs. EDTA-treated cells).

### Adhesion assay

Adhesion assays were performed based on the method of Stepanović et al. (2007), with some modifications. Bacterial cultures were centrifuged at 2151 × g for 5 min at 24 ° C, washed twice with phosphate-buffered saline (PBS, pH 7.2, Sigma-Aldrich, Inc., USA), and diluted in LB broth (BD Difco, USA) to an optical density of 0.5 McFarland units (approximately 1.5 × 10^8^ cells/ml) at 565 nm using a densitometer (DEN-1B, Biosan, Latvia). A 12-well plate (Genoplast, Poland) was inoculated in triplicate with the bacterial suspensions. After a 1 h incubation at 37 ° C without agitation, each well was gently washed three times with PBS (pH 7.2, Sigma-Aldrich, Inc., USA) and dried at 60 ° C for 1 h. Adherent bacteria were stained with 0.5% crystal violet (Sigma-Aldrich, Inc., USA), and absorbance at 580 nm of the solubilized stain in ethanol was measured using a microplate reader (BIOLOG, BioTeK Instruments, Inc., USA). All experiments were performed in triplicate.

### Aggregation assay

Aggregation assays were performed as described by Eboigbodin and Biggs (2008). Bacterial cells were washed twice and resuspended in 0.9% NaCl (pH 7.0). The optical density at 600 nm (OD_600,0_) of the suspension was adjusted to approximately 0.6. Then, 2 mL of the suspension was transferred into a cuvette. As the cells aggregated and settled, the optical density (OD_600,t_) was measured every hour up to 6 h. All experiments were performed in triplicate. The percentage of aggregation was calculated according to the following equation:

aggregation % = [(OD_600,0_ – OD_600,t_)/OD_600,0_] x 100 (1).

### Statistics

All results are represented as mean ± standard deviation (SD). Statistical comparisons were performed using the non-parametric Kruskal-Wallis one-way analysis of variance on ranks or the Mann-Whitney rank sum test. Subpopulations detection was conducted using k-means cluster analysis, with the optimal number of clusters determined by the elbow method. One-way ANOVA and Newman-Keuls *post hoc* test were used for multiple comparisons of means to detect statistically significant differences between cells. Data were analyzed using Statistica Software (StatSoft, USA).

## Results

### Effect of EDTA on the bacterial cell surface

**Fig. 1:**
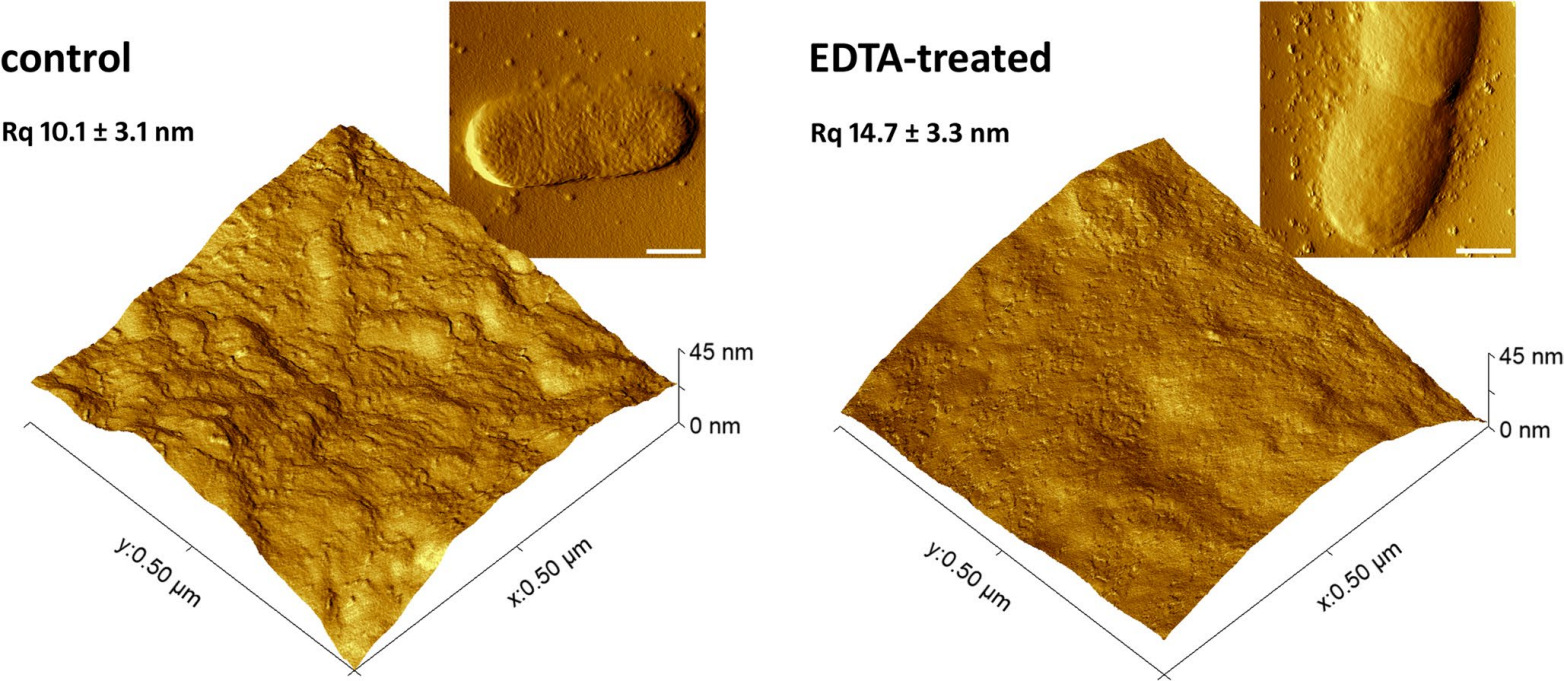
Three-dimensional topographic and amplitude images of *E. coli* control and EDTA-treated cells. Surface structures scanned over a fixed area of 500 × 500 nm^2^. Inset: corresponding amplitude images of bacterial cells. Scale bar: 500 nm. The root mean square roughness (Rq) was measured for a selected area of the bacterial surface from the topographic images

AFM imaging revealed alterations in the cell surface induced by EDTA treatment (Fig. 1). While the bacteria retained their native cylindrical shape and remained similar in size, the outer membrane became smoother and featureless. The protrusions composed of LPS bundles, observed on the membrane surface of native cells, disappeared after treatment. The outer membrane exhibited numerous shallow, irregularly shaped pits and a rougher surface texture compared to untreated cells. Surface roughness (Rq) increased from 10.1 ± 3.1 nm to 14.7 ± 3.3 nm (difference was not statistically significant, *P* = 0.055). Additionally, some control and EDTA-treated cells were surrounded by extracellular material, likely outer membrane vesicles (OMVs) and released LPS molecules. This observation further supports that cell viability was maintained, since OMVs are typically secreted during bacterial growth, particularly at the end of the logarithmic phase.

### Heterogeneity in adhesive and mechanical properties of the treated cells

**Fig. 2:**
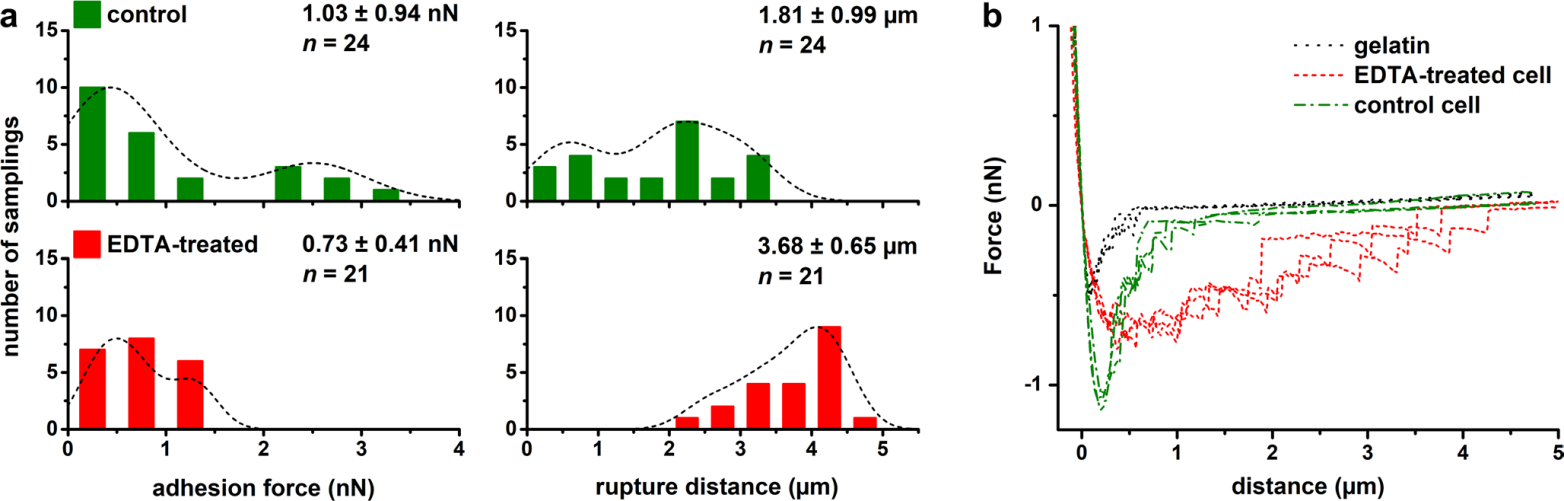
Histograms of adhesion force and rupture distance with corresponding distribution curves (**a**) and representative retraction force-distance curves (**b**) obtained for *E. coli* control and EDTA-treated cells. Kernel density estimation was applied to fit the distributions of the experimental points, i.e. single bacteria. Mean and standard deviation are presented with the number of analyzed bacterial cells (n)

**Fig. 3:**
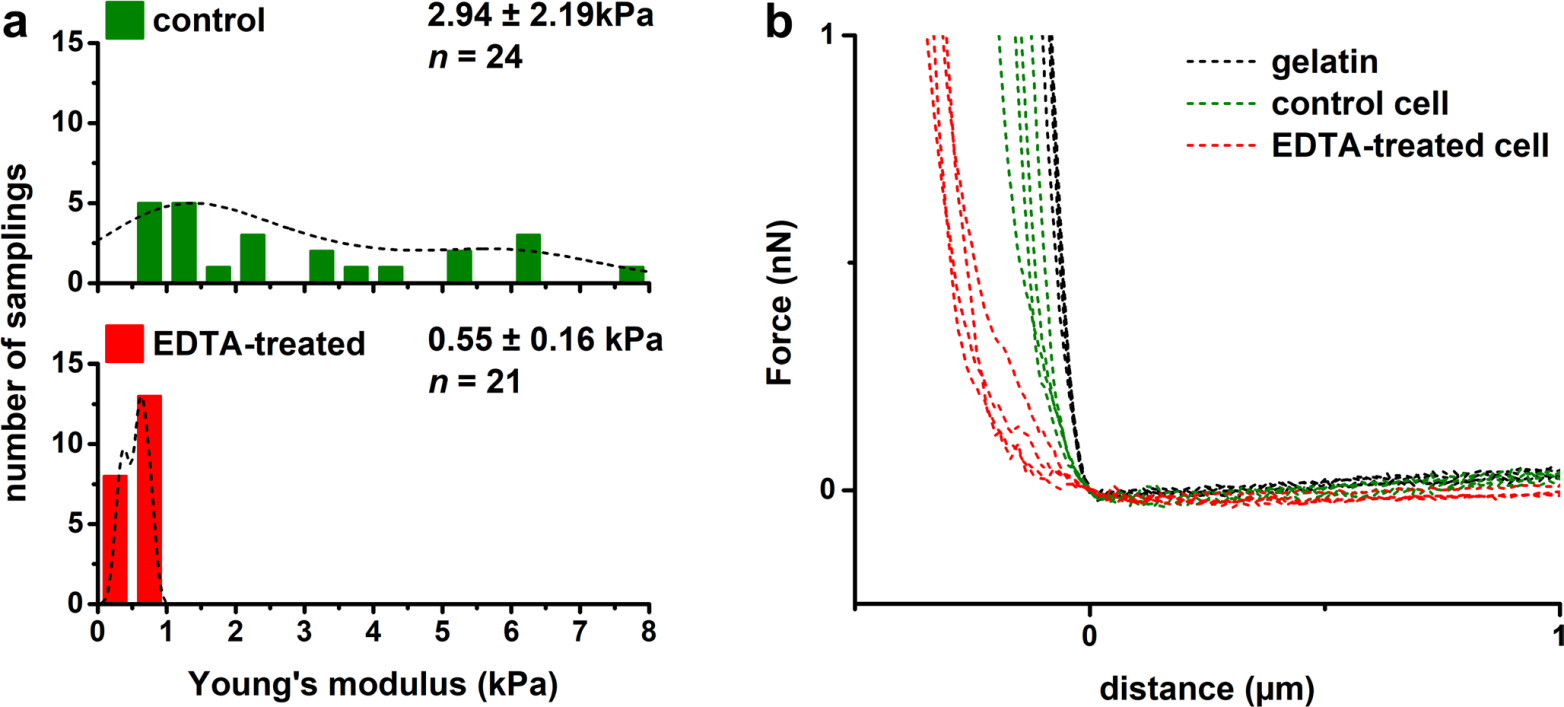
Histograms of Young’s modulus with corresponding distribution curves (**a**) and representative approach force-distance curves (**b**) obtained for *E. coli* control and EDTA-treated cells. Kernel density estimation was applied to fit the distributions of the experimental points, i.e. single bacteria. Mean and standard deviation are presented with the number of analyzed bacterial cells (n)

**Fig. 4:**
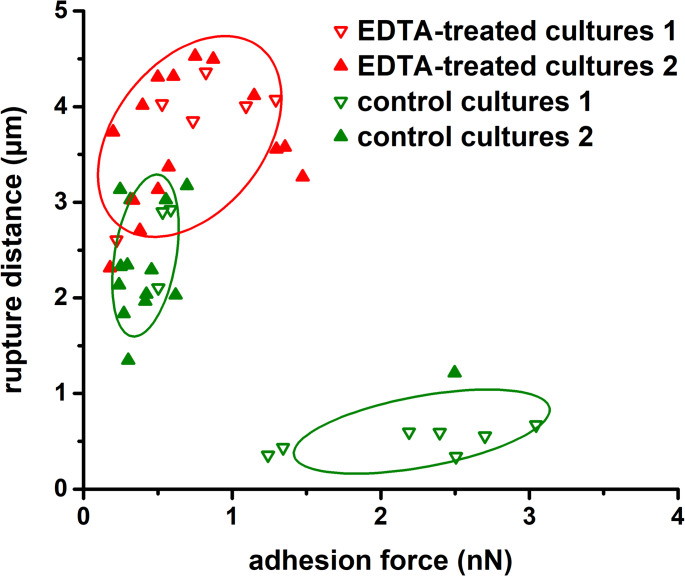
Heterogeneity in adhesive properties of *E. coli* cells from two independent cultures: control and EDTA-treated. Ellipses indicate subpopulation clusters with a 95% confidence level. Each point represents the mean value from force-distance curves measured on a single cell

Cells from both independent cultures exhibited variability in adhesive and mechanical properties. Histograms with kernel density estimation (KDE) curves for control cells showed two local maxima, which can be attributed to heterogeneity within the bacterial population (Figs. 2A and 3A). Cluster analysis based on Young’s modulus, adhesion forces, and rupture distance, i.e. the extension length during probe detachment of individual cells, identified two distinct *E. coli* subpopulations in each culture (Fig. 4). Subpopulation 1 was characterized by a higher adhesion forces, shorter interaction length, higher stiffness (as indicated by Young’s modulus), and a higher heterogeneity index compared to subpopulation 2 (Table 1).

EDTA treatment significantly altered the adhesive and mechanical properties of *E. coli*. Cells with released LPS molecules showed more homogeneous surface properties and lower heterogeneity than control cells (Figs. 2A and 3A). Adhesion forces were reduced upon LPS removal, whereas rupture distance increased significantly (Fig. 2). Force-distance curves of treated cells exhibited a significantly greater number of adhesive events and a reduced slope with subtle irregularities compared to control cells, which may indicate a weakening of the bacterial cell wall (Figs. 2B and 3B). The mean adhesion force decreased from 1.03 ± 0.94 nN in control cells to 0.73 ± 0.41 nN in EDTA-treated cells. In contrast, mean rupture distance increased from 1.81 ± 0.99 μm to 3.68 ± 0.65 μm (difference was statistically significant, at *P* < 0.001) (Fig. 2A). A significant decrease in cell stiffness was also observed, with Young’s modulus reduced from 2.94 ± 2.19 kPa to 0.55 ± 0.16 kPa (difference was statistically significant, *P* < 0.001) (Fig. 3). Additionally, statistically significant pairwise differences in adhesion forces and Young’s modulus were more frequent in control cells compared to EDTA-treated cells: 53% vs. 44% for adhesion forces and 76% vs. 6% for Young’s modulus, respectively (ANOVA at *P* < 0.01) (Fig. S2).

**Fig. 5:**
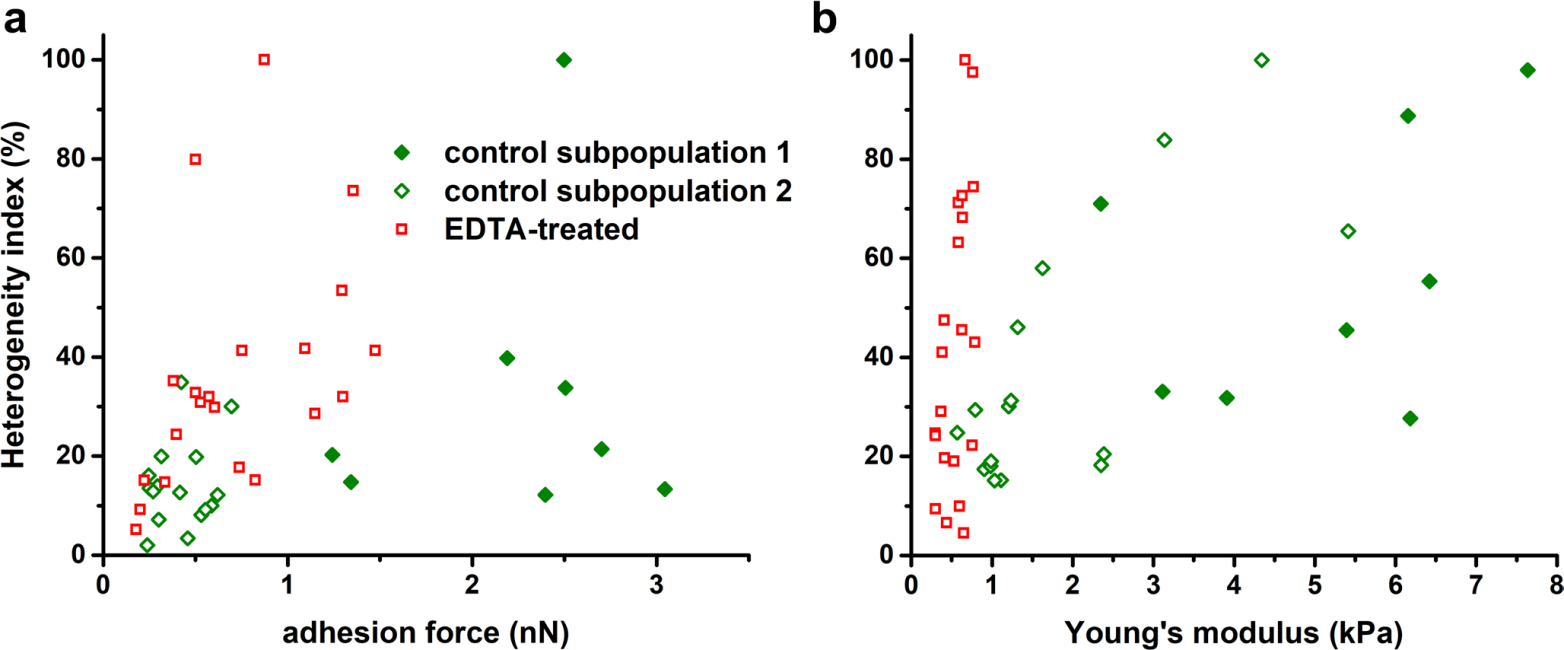
Relationship between heterogeneity index (HI) and adhesion force (**a**) or Young’s modulus (**b**) for *E.* coli control and EDTA-treated cells. Each point represents the mean value from a force-distance curves recorded on an individual cell

LPS removal resulted in a marked reduction in heterogeneity, as evidenced by narrower data distribution and decreased coefficient of variation (reduction by 40%). The distinction between subpopulations also became less pronounced (Figs. 2 and 3). Notably, as shown in the scatter plot (Fig. 4) and summarized in Table 1, cells with a strongly adherent and stiff phenotype (subpopulation 1) were no longer present following treatment. The intergroup heterogeneity index decreased by 54% and 92% for the adhesive and elastic properties, respectively. Intragroup HI values for adhesion were 20% in control cells and 41% in EDTA-treated cells. Among *E. coli*, only one untreated cell exhibited high heterogeneity (HI > 50%), whereas four EDTA-treated cells reached this threshold (Fig. 5A). For elasticity, intragroup HI values were 42% in both control and EDTA-treated groups, with seven cells in each showing high heterogeneity (Fig. 5B).

In general, the heterogeneity index tended to increase with higher adhesion force or Young’s modulus in individual cells. However, no consistent analytical relationship could be established to describe the trends observed in the scatter plot (Fig. 5).

### Adhesion and aggregation assays

**Fig. 6:**
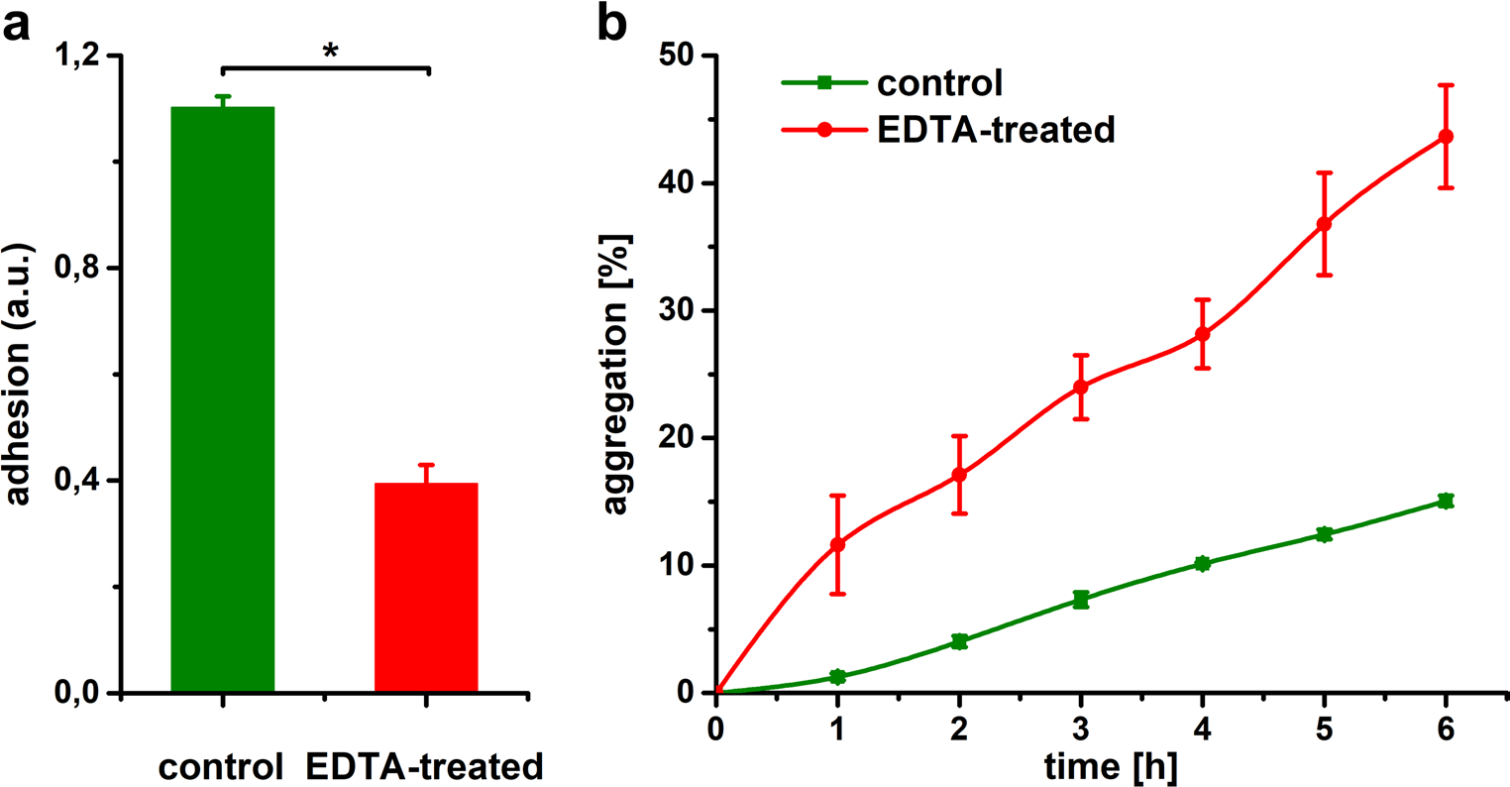
Adhesion ability (**a**) and aggregation kinetics (**b**) of *E.* coli control and EDTA-treated cells at the population level. Boxes represent mean and standard deviation. Statistically significant differences (Mann-Whitney rank sum test, *P* < 0.01) are denoted by a mark (*)

At the population level, heterogeneity in adhesion properties (measured by coefficient of variation) was substantially lower than that observed at the single-cell level. The ability of bacteria to adhere was reduced by 64% following LPS release (Fig. 6A), while the aggregation potential of EDTA-treated cells was significantly higher than that of native cells and increased progressively over time (Fig. 6B). The results were statistically significant (*P* < 0.01).

## Discussion

The survival of prokaryotes depends on the cell’s ability to respond to environmental pressures, including mechanical forces. Bacteria can modify their surface structure and shape, thereby adapting biophysical properties that originate from surface-associated molecules such as lipopolysaccharides, peptidoglycans, proteins, and fimbrial or non-fimbrial appendages (Araújo et al. 2019).

In this study, we demonstrate that lipopolysaccharide-mediated diversity in the outer membrane significantly contributes to phenotypic heterogeneity in bacterial adhesion and mechanics at the single-cell level. Our results revealed that clonal *E. coli* populations exhibit marked variability in biophysical properties in both independent cultures. We identified two subpopulations with distinct adhesive and mechanical characteristics. This phenotypic heterogeneity became less pronounced after partial LPS removal. The population no longer contained cells with a strongly adherent and stiff phenotype, suggesting that structural LPS diversity plays an important role in modulating bacterial adhesion to surfaces and contributes to cell stiffness, along with peptidoglycan. Furthermore, we demonstrated that bacterial populations exhibiting heterogeneous surface properties may possess a selective advantage compared to those with uniform biophysical characteristics.

To our knowledge, this is the first study in which adhesion forces and elasticity were simultaneously measured at the single-cell level in a statistically significant bacterial population. We focused on heterogeneity among cells within the population, rather than heterogeneity of individual cell surfaces, which is typically caused by the spatial organization of molecules in the outer membrane. For this purpose, a silica microbead was mounted on a tipless cantilever to probe the interactions between individual immobilized live bacteria and the AFM probe. Silica was chosen as a model soil-like surface due to its surface charge, which is similar to that of soil particles (Park and Abu-Lail 2011), and its ecological relevance as a common environmental component (Chenu and Stotzky 2002). A key advantage of using a colloidal probe is the ability to probe the entire bacterial surface under a controlled mechanical load, enabling rapid characterization of cell surface properties through a single measurement performed on multiple cells. This is particularly relevant, as surface-attached bacteria in natural environments are exposed to physical stresses (Park and Abu-Lail 2011; Viela et al. 2020). In force spectroscopy, the loading force also contribute to cell deformation (Chen et al. 2014). To minimize this effect, we applied a loading force of up to 500 pN, which mainly deforms the cell envelope of the immobilized bacteria. Moreover, colloidal probes provide a more comprehensive characterization of cell mechanical properties (Araújo et al. 2019), and reduce the heterogeneity typically observed in adhesion measurements using nanoscale tips (Park and Abu-Lail 2011).

The first critical decision in our study concerned the method used for outer membrane modification. EDTA was chosen due to its well-characterized mode of action (Clifton et al. 2015; Finnegan and Percival 2015), antimicrobial and antibiofilm activities, and ability to enhance the efficacy of various antimicrobial agents (Liu et al. 2018). EDTA removes a substantial portion of LPS molecules (approximately 50–80%), inducing outer membrane remodeling (Clifton et al. 2015) without significantly affecting cell viability, growth rate, or protein synthesis capacity (Abu-Lail and Camesano 2003; Chen et al. 2009; Pandur et al. 2022). However, EDTA treatment may also induce additional alterations in the outer membrane, including increased permeability, protein removal, and impaired motility due to effects on flagella (Abu-Lail and Camesano 2003). Other antibacterial agents such as polymyxin (Torcato et al. 2013; Paracini et al. 2022), rhamnolipids (Gdaniec et al. 2022), or lactoferrin (Fux et al. 2023) also interact with the outer membrane, inducing effects comparable to those of EDTA. Due to its widespread use in various industries (Finnegan and Percival 2015; Evstatiev et al. 2021) and low biodegradability, EDTA accumulates in aquatic and terrestrial environment (Bloem et al. 2017). The second important decision was to use standard axenic cultures instead of synchronized cultures, in order to preserve natural population variability. In our previous research, axenic cultures of Gram-negative bacteria exhibited higher heterogeneity than Gram-positive species, regardless of whether measurements were conducted at several discrete points (AFM tip) (Laskowski et al. 2018) or across the entire bacterial surface (colloidal probe) (Laskowski et al. 2021).

Our results, based on both single-cell and population level measurements, revealed that LPS removal by EDTA substantially altered *E. coli* cell surface, affecting the adhesive and mechanical properties. Similar alterations in the outer membrane following LPS removal have been reported previously (Amro et al. 2000; Alakomi et al. 2006; Chen et al. 2009). However, the studies investigating the effect of EDTA on cell surface roughness have reported inconclusive results. Amro et al. (2000) observed an increased roughness (consistent with our findings), whereas Chen et al. (2009) reported a decrease. These discrepancies may result from differences in LPS removal procedure and using cantilevers with different tip radii.

Cells with partially removed LPS exhibited more homogeneous surface properties, characterized by reduced adhesion forces, extended interaction distances with more frequent adhesive events, and lower stiffness (as indicated by Young’s modulus) relative to control cell. These findings are consistent with previous studies demonstrating that EDTA-mediated LPS removal from *E. coli* reduces cell adhesion to AFM tip (Abu-Lail and Camesano 2003) and decreases cell rigidity (Chen et al. 2009; Rojas et al. 2018). Moreover, reduction in cell stiffness is dependent on EDTA concentration (Rojas et al. 2018). Similarly, Strauss et al. (2009) showed that *E. coli* strains with longer LPS chains exhibited stronger adhesion force, but only in strains expressing O-antigen. In contrast to our results, Abu-Lail and Camesano (2003) reported significant decreases in rupture lengths following EDTA treatment. This discrepancy likely reflects differences in AFM probes geometries (sharp vs. colloidal), as contact area significantly influences force spectroscopy results (Vadillo-Rodriguez et al. 2008; Chen et al. 2014; Fu et al. 2024; Gnanachandran et al. 2025). Chelation of divalent cations by EDTA further induces LPS molecules translocation (flip-flop) into the inner bilayer leaflet, exposing inner membrane phospholipids at the outer leaflet and creating phospholipid-rich patches. This disruption of bilayer asymmetry increases membrane permeability, enhances cell surface hydrophobicity, and decreases outer membrane stiffness (Clifton et al. 2015; Finnegan and Percival 2015; Pandur et al. 2022; Sun et al. 2022).

Although several studies have reported broad data distributions reflecting the heterogeneity of macromolecules on the bacterial surfaces, few have identified distinct subpopulations within the bacterial cultures (Park and Abu-Lail 2011; El-Kirat-Chatel et al. 2017; Tamayo et al. 2020). In contrast, our study revealed that phenotypic heterogeneity was significantly reduced after LPS removal, with diminished distinction between subpopulations. This observed heterogeneity within the control population may reflect several factors, including the presence of cells in different growth phases (logarithmic vs. stationary), natural variability in LPS structure, or the potential cessation of O-antigen biosynthesis by certain cells to avoid the energy-intensive process. During transition to the stationary phase, the cell envelope of *E. coli* undergoes alterations to enhance stress resistance. The total protein content of the outer membrane decreases, while the cross-linking of lipoproteins between the OM and PG increases (Mitchell et al. 2016). Consequence, cells in the stationary growth phase exhibit a strengthened permeability barrier, rendering them less sensitive to EDTA (Pandur et al. 2022).

Cell-to-cell variation can be attributed, in part to the structural heterogeneity of lipopolysaccharides. LPS exhibits considerable variability in terms of both surface coverage density and local spatial distribution. A single bacterial cell contains roughly 3.5 million LPS molecules, which cover about 75% of the cell surface and are organized into bundles of 600–3500 molecules (Saini and Wood 2020; Fux et al. 2023). LPS is composed of three structurally distinct domains: lipid A, a hydrophobic glycolipid; a highly anionic oligosaccharide core; and the hydrophilic O-antigen. The O-antigen exhibits substantial variability in chain length and branching, even within a single cell and among cells from the same bacterial culture (Ebbensgaard et al. 2018; Saini and Wood 2020; Paracini et al. 2022; Fux et al. 2023). Under conditions of weak or absent selection pressure, some bacteria may lose the ability to synthesize surface antigens, whereas adaptation to harsh environments can induce alterations in LPS biosynthesis. Smooth-to-rough mutations are usually irreversible transitions that involve either progressive shortening or complete cessation of O-chain synthesis (Lukácová et al. 2008; King et al. 2009). Even the same wild-type strain (e.g. *E. coli* O111) may express smooth, semi-rough and rough LPS phenotypes (Pupo et al. 2013). In general, smooth LPS contains a considerable fraction (up to 60% molar fraction) of short LPS molecules bearing either no or a single O-antigen repeat unit (Paracini et al. 2022). For example, *E. coli* JM109 synthesizes O-antigen chains ranging from 1 to 26 repeat units, with only about half of the LPS molecules contain more than 3 units (Saini and Wood 2020). The smooth phenotype predominates in nature, and most clinical isolate of *E. coli* express smooth LPS (Ebbensgaard et al. 2018; Fux et al. 2023). In contrast, most laboratory adapted strains (e.g. *E. coli* K12) have lost the ability to synthesize the O-antigen, resulting in rough LPS (Paracini et al. 2022). The strain used in this study, *E. coli* ATCC 25922, possesses smooth LPS (Torcato et al. 2013).

This remarkable structural diversity, along with the emergence of heterogeneous bacterial populations, is driven by a variety of factors (Paracini et al. 2022; Fux et al. 2023). One key source is stochastic gene expression, which includes processes such as transcription, translation, and post-translational modifications. Other mechanisms include phase variation, epigenetic regulation, and the differential responses of individual bacteria to environmental signals (Avery 2006; Carlquist et al. 2012; Davis and Isberg 2016). Phase variation is particularly significant, as it enables rapid and reversible alteration of cell surface structures, including modifying antigenic specificity, for instance, by altering LPS (Avery 2006; van der Woude 2011; Davis and Isberg 2016). Importantly, such heterogeneity can emerge even in a constant microenvironments (Carlquist et al. 2012).

Therefore, we conclude that EDTA-induced modifications to the outer membrane diminishes the natural structural diversity of the cell surface, thereby reducing phenotypic heterogeneity within the population and altering the biophysical properties of *E. coli*. The release of LPS and capsular polysaccharides primarily contributes to reduced adhesion forces and overall cell rigidity. However, the partial removal of membrane proteins also significantly impacted elasticity. The outer membrane contains two major classes of proteins: outer membrane proteins (OMPs) and lipoproteins. OMPs are highly abundant, with approximately 0.5 million per *E. coli* cell, and are organized into islands that cover around 70% of the cell surface, which highlight their significant role in maintaining the physical properties of the outer membrane. Meanwhile, membrane-anchored lipoproteins contribute to various cellular pathways, with OmpA, Pal and Lpp lipoproteins playing a key role in preserving cell rigidity (Sun et al. 2022).

Our force spectroscopy data are consistent with the adhesion assay, which similarly showed reduced adhesion capacity of the EDTA-treated cells. Importantly, we observed significantly lower population level variability compared to single-cell measurements. These results are consistent with previous studies demonstrating substantial reductions in bacterial adhesion to various surfaces following LPS removal (Abu-Lail and Camesano 2003; Alakomi et al. 2006; Hori and Matsumoto 2010). We also observed enhanced aggregation in treated cells. Nakao et al. (2012) reported that the *E. coli* deep rough LPS mutant exhibits enhanced autoaggregation accompanied by increased cell surface hydrophobicity. Furthermore, LPS released into the environment may promote aggregation of treated cells by acting analogously to the extracellular polymeric substance (EPS) (Zhang et al. 2014). LPS molecules, which are released during cell division or death, can accumulate in the environment, and self-assemble into diverse supramolecular structures (Fux et al. 2023).

## Conclusion

In conclusion, our combined analyses at the single-cell and population levels demonstrate that the structural diversity of lipopolysaccharides is a key determinant of phenotypic heterogeneity and plays a crucial role in modulating the biophysical properties of *E. coli*. The chemical removal of LPS substantially altered the bacterial cell surface, reducing both cell adhesion and elasticity. Disorganization of the outer membrane by EDTA also markedly diminished the natural structural heterogeneity of the cell envelope, resulting in a more uniform phenotype within the clonal population. Our findings highlight the functional importance of LPS structural diversity in regulating bacterial surface interactions and mechanical behavior, with significant implications for understanding bacterial adhesion, environmental adaptation, and responses to antimicrobial stressors. AFM-based force spectroscopy with colloidal probes provides a powerful platform for quantifying cell-to-cell variability in biophysical properties. Furthermore, this approach enables the targeted study of specific ligand-receptor interactions through probe functionalization, as well as the analysis of non-specific forces using probes composed of different materials.

**Table 1:** Summary of mechanical and adhesive properties and heterogeneity indices for *E. coli* control and EDTA-treated cells

*E. coli*		Number of cells	Adhesion forces (nN)	Rupture distance (µm)	Young’s modulus (kPa)	Intergroup HI (%)
Control	Subpopulation 1	8	2.24 ± 0.64 a	0.59 ± 0.28 b	5.15 ± 1.83 a	100
	Subpopulation 2	16	0.42 ± 0.15 b	2.41 ± 0.55 c	1.84 ± 1.38 b	17
EDTA-treated		21	0.73 ± 0.41 b	3.68 ± 0.65 a	0.55 ± 0.16 b	40

Statistically significant differences (one-way ANOVA and Newman-Keuls *post hoc* test, P < 0.001) are denoted by different letters. Mean and standard deviation are presented.

## Supplementary Information

Below is the link to the electronic supplementary material.

Supplementary Material 1

## Data Availability

The datasets generated and analyzed during the current study are available from the corresponding author on reasonable request.
