# Supplementary material for: Single-cell analysis of lipopolysaccharide-mediated heterogeneity in Escherichia coli adhesion and mechanics
Source: Arch Microbiol. 2025 Oct 18;207(12):321. doi: 10.1007/s00203-025-04529-3 (PMC12535489; doi:10.1007/s00203-025-04529-3)
Supplement: Supplementary file 1 — Supplementary Material 1 [file 203_2025_4529_MOESM1_ESM.pdf]

Single-cell analysis of lipopolysaccharide-mediated heterogeneity in *Escherichia coli* adhesion and mechanics

Archives of Microbiology Journal

Dariusz Laskowski[a*], Janusz Strzelecki[b]

[a] Department of Microbiology, Faculty of Biological and Veterinary Sciences, Nicolaus Copernicus University in Toruń, Lwowska 1, 87-100 Toruń, Poland

[b] Institute of Physics, Faculty of Physics, Astronomy and Informatics, Nicolaus Copernicus University in Toruń, Grudziądzka 5, 87-100 Toruń, Poland

Corresponding author*

Dariusz Laskowski

laskosd@umk.pl

Orcid: 0000-0002-2362-8904

Janusz Strzelecki

Orcid: 0000-0002-7830-7315

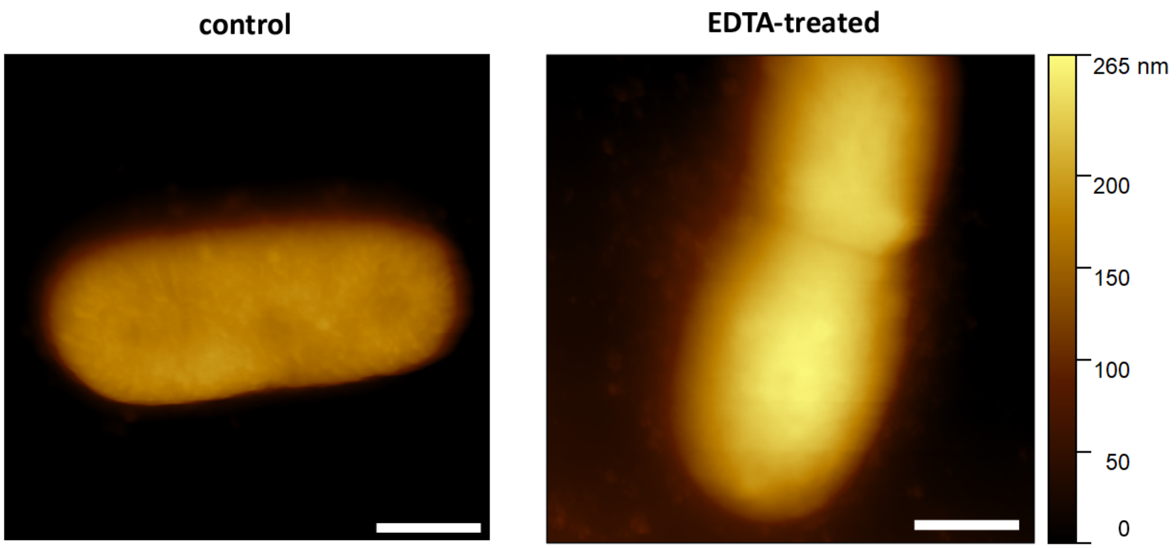

Fig. S1 Topography images of *E. coli* control and EDTA-treated cells. Scale bar: 500 nm. Topography images were used to quantify surface roughness (Rq)

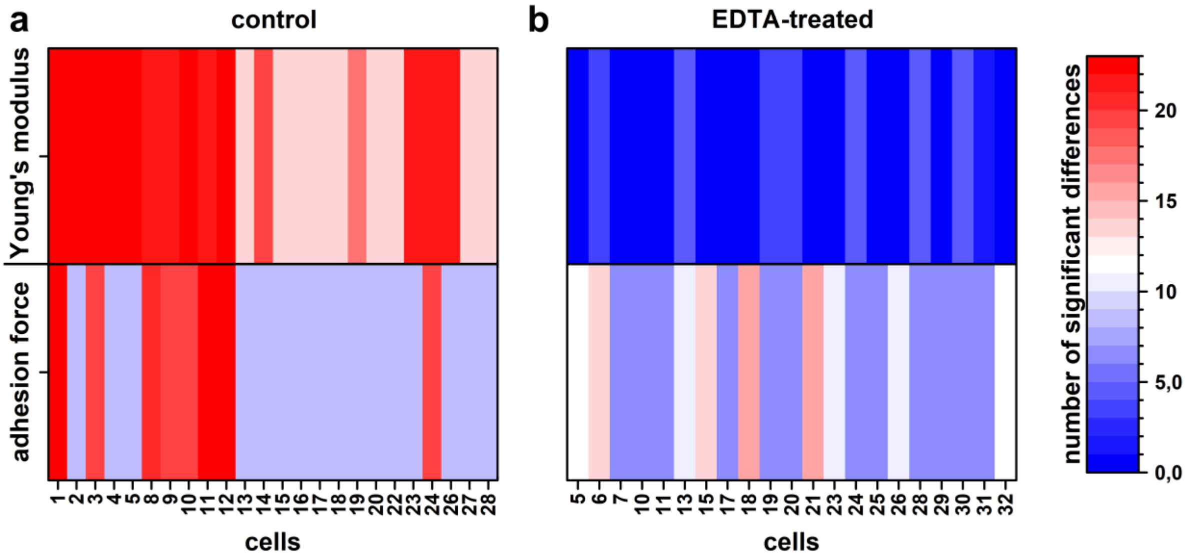

Fig. S2 Heatmaps showing statistically significant pairwise differences (one-way ANOVA with Newman-Keuls *post hoc* test, $P < 0.01$) in adhesion forces and Young's modulus for *E. coli* control (a) and EDTA-treated cells (b)
